# *De Novo* Occurrence of a Variant in *ARL3* and Apparent Autosomal Dominant Transmission of Retinitis Pigmentosa

**DOI:** 10.1371/journal.pone.0150944

**Published:** 2016-03-10

**Authors:** Samuel P. Strom, Michael J. Clark, Ariadna Martinez, Sarah Garcia, Amira A. Abelazeem, Anna Matynia, Sachin Parikh, Lori S. Sullivan, Sara J. Bowne, Stephen P. Daiger, Michael B. Gorin

**Affiliations:** 1 Department of Pathology and Laboratory Medicine, University of California Los Angeles, Los Angeles, California, United States of America; 2 Personalis Inc., Menlo Park, California, United States of America; 3 Department of Cardiology, Cedars-Sinai Medical Center, Los Angeles, California, United States of America; 4 Research Institute of Ophthalmology, Cairo, Egypt; 5 Jules Stein Eye Institute and Department of Ophthalmology, University of California Los Angeles, Los Angeles, California, United States of America; 6 Human Genetics Center, University of Texas Health Science Center, Houston, Texas, United States of America; Hadassah-Hebrew University Medical Center, ISRAEL

## Abstract

**Background:**

Retinitis pigmentosa is a phenotype with diverse genetic causes. Due to this genetic heterogeneity, genome-wide identification and analysis of protein-altering DNA variants by exome sequencing is a powerful tool for novel variant and disease gene discovery. In this study, exome sequencing analysis was used to search for potentially causal DNA variants in a two-generation pedigree with apparent dominant retinitis pigmentosa.

**Methods:**

Variant identification and analysis of three affected members (mother and two affected offspring) was performed via exome sequencing. Parental samples of the index case were used to establish inheritance. Follow-up testing of 94 additional retinitis pigmentosa pedigrees was performed via retrospective analysis or Sanger sequencing.

**Results and Conclusions:**

A total of 136 high quality coding variants in 123 genes were identified which are consistent with autosomal dominant disease. Of these, one of the strongest genetic and functional candidates is a c.269A>G (p.Tyr90Cys) variant in *ARL3*. Follow-up testing established that this variant occurred *de novo* in the index case. No additional putative causal variants in *ARL3* were identified in the follow-up cohort, suggesting that if *ARL3* variants can cause adRP it is an extremely rare phenomenon.

## Introduction

Retinitis pigmentosa (RP) [MIM: 268000] is a clinical finding defined by peripheral vision loss due to impaired retinal function which affects approximately 1:3,000–1:5,000 individuals [1, 2]. The genetic basis of RP is highly heterogeneous, with at least fifty genes identified to date as associated with non-syndromic RP [3–8]. Most known forms of inheritance have been demonstrated for RP, including single-gene autosomal recessive, autosomal dominant, and X-linked inheritance and digenic inheritance [9–11].

Due to this extreme heterogeneity, massively parallel DNA sequencing has become an integral part of both clinical diagnostics and research of the genetic basis of RP and other retinal dystrophies [6, 10, 12–17]. Gene panels are now used clinically to identify variants in known retinal dystrophy genes, with a success rate of approximately 20–40% [6, 16]. Exome sequencing, the genome-wide identification and analysis of DNA variants resulting in a predicted change in any known protein-coding gene, has been particularly powerful for variant and disease gene discovery for retinal dystrophies [12, 17] and across nearly all areas of genetics [18–21]. These tools provide a cost-effective measure to ending the diagnostic odyssey so often endured in medical genetics.

**Fig 1:**
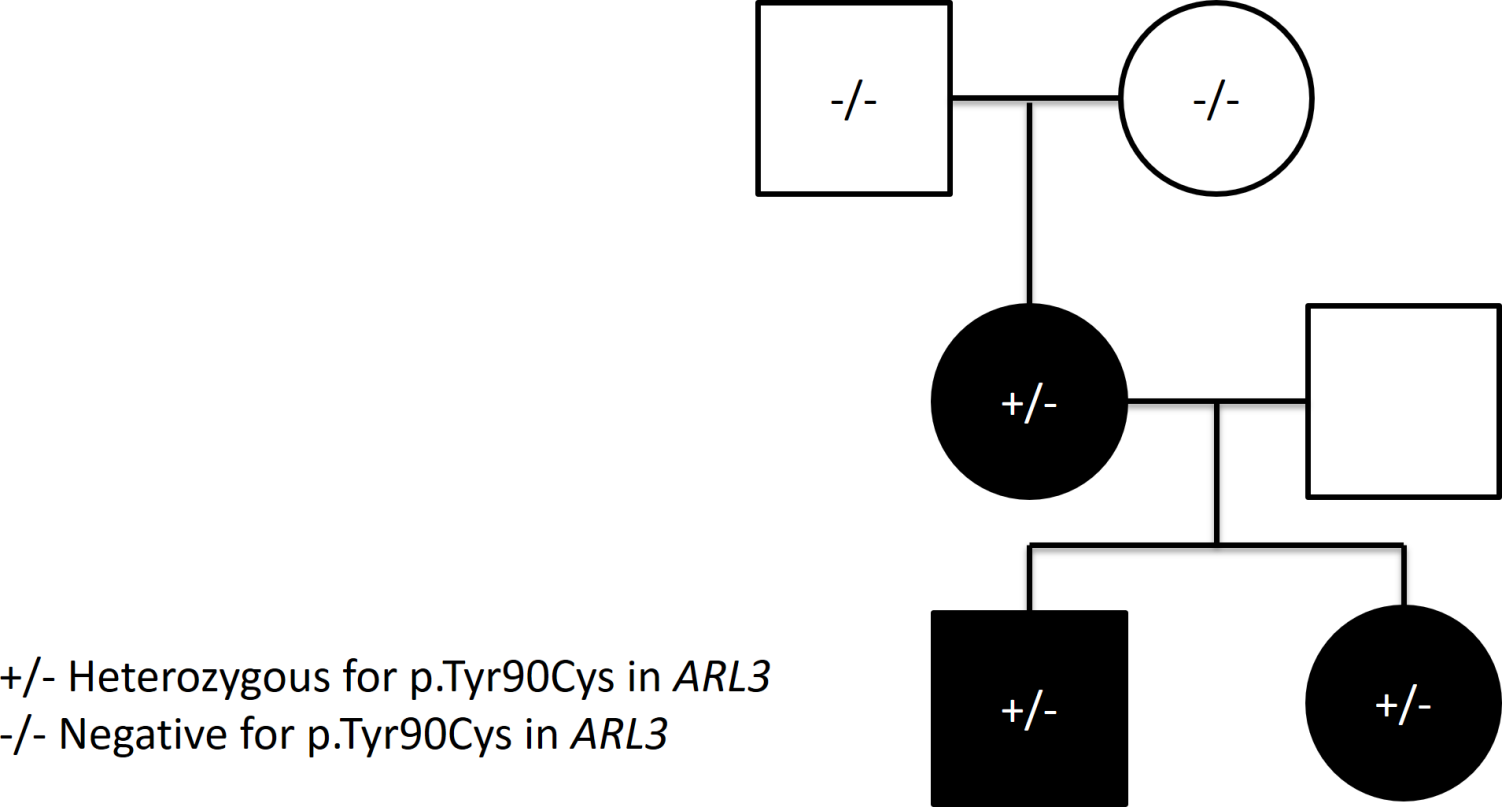
Pedigree of family with apparent autosomal dominant retinitis pigmentosa. *De novo* occurrence of a variant in *ARL3* in an individual with retinitis pigmentosa and subsequent transmission to affected offspring.

Pedigree RP02 consists of a four-person nuclear family of reported European Caucasian descent. Three individuals in this pedigree are affected with non-syndromic retinitis pigmentosa (Fig 1). As the trait is observed in the mother and one child of each gender, autosomal dominant inheritance was considered a strong possibility. Given the large number of genes and variants which can cause autosomal dominant retinitis pigmentosa (adRP) [7, 8, 22], exome sequencing was performed on the three affected individuals to identify possible causal DNA variants.

As each individual exome contains 20,000–25,000 DNA variants compared to the human genome reference sequence, a series of bioninformatic analyses were performed first to identify variants within known retinal disease genes and then, if negative, to continue the search for potentially novel disease genes.

## Materials and Methods

### Patient Samples

This study was conducted within an Institutional Review Board (IRB)-approved human subjects research program at the University of California Los Angeles (IRB#11–000020, "Genetic Studies in Inherited Ocular Disorders") and the study was conducted in accordance with regulations of the Health Insurance Portability and Accountability Act of 1996 (HIPAA). All three individuals enrolled in the Genetics research study and signed a consent form. The UCLA HIPAA consent form specifies the use of the information for publications and research presentations. DNA samples from the nuclear family were obtained from whole blood specimens using standard protocols. DNA samples from parental samples of the index case were collected and extracted using Oragene DNA Saliva kits (DNA Genotek Inc., Ottowa, Ontario, Canada).

#### Retrospective case report

Mother: At 58 years old at presented with difficulty reading with mild decreased visual acuity and bilateral visual field concentric constriction. She has a history of cataract surgery at age 41 years. Her visual acuity measured as 20/60 in the right eye and 20/80 in the left. Intraocular pressures (IOP) were 12 and 10 for the right and left eye respectively. Visual fields were “central islands” bilaterally, and the retina showed the classic pattern of mid-peripheral pattern of pigmentation with attenuated vessels. Optical coherence tomography (OCT) showed no edema. No other relevant medical problems were reported. Previous genetic testing included mutation screening in the following genes: *CRX*, *PRPH2*, *RP1* and *IMPDH1*. All were negative.

Daughter: At 27-years old had late onset nyctalopia, decreased vision with difficulty of reading and driving, flashes and floaters for several years and progressive loss of peripheral vision. She has also medical history of difficulties with gait, headaches, difficulties with balance, increased urinary frequency and severe ankle swelling (partially responsive to Lasix, possibly familial lymphedema).

Visual acuity for right and left eyes was 20/80-1 20/60-2. IOP right and left were 13 and 14. Bilateral visual field constriction was observed. A posterior subcapsular cataract (PSC) was identified in the left eye only. Intraocular lens (IOC) noted in the right eye only. Fundus examination revealed vitreous floater in the right eye only. Bilateral attenuation of retinal vessels and bone-spicules were observed in the periphery. OCT showed cystoid macular edema.

Son.: At 30 years-old presented with retinitis pigmentosa and related cystoid macular edema. The patient continues to be very bothered by flashes of light that are severe enough to be almost disabling. Also he has floaters and progressive loss of peripheral vision. The patient had different surgical procedures as Lasik OU (2003), cataract removal OD (2008), Yag, strabismus for exotropia 15–20Δ at 12 and 14 years old. Medical history

Visual acuity for right and left eyes was 20/20 and OS: 20/25+2. IOP left and right were 13 and 14. Both visual fields were constricted. A posterior subcapsular cataract PSC was identified in the left eye only and IOC noted in the right eye only, the same pattern as in his sister. No other relevant medical problems were reported.

### Exome Sequencing

Exome sequencing was performed using the Accuracy and Enhanced Content Exome (ACE) pipeline developed by Personalis (Menlo Park, CA). This pipeline is a clinically validated and published protocol including all stages of analysis including: library preparation, target capture, sequencing, alignment, variant calling, and variant annotation [23]. This methodology detects single nucleotide variants and small insertions and deletions up to approximately 20 nucleotides in length. Larger genetic changes such as deletions, duplications, and copy-neutral structural rearrangements were not analyzed.

The following *in silico* tools were used to predict the likely impact on protein function of observed DNA variants: LRT, PolyPhen2 [24], SIFT [25], MutationTaster [26], and intra-species conservation (GERP, PhyloP, PhastCons).

### Variant Filtering

Variants were filtered for quality by removing any variant with a VCF flag of anything except “PASS” and/or having a QUAL score <500. The maximum of 1000 Genomes and Exome Sequencing Project (ESP5400) used as the estimate of minor allele frequency (MAF). Variants below 1% MAF were considered “rare” while variants > = 1% were filtered out of analysis. Synonymous and intronic variants not known to cause splice defects were also removed during filtering (see S1 Table and S2 Table).

### Protein-Protein Interaction Prediction

The predicted impact of candidate variants on protein-protein interactions were estimated using mCSM [27].

### Sanger Confirmation and Follow-Up

PCR amplification followed by dideoxy capillary electrophoresis DNA sequencing was performed to validate the variant in *ARL3* (S1 Fig).Primer sequences are listed in S3 Table. Parentage of the grandparents was confirmed by the UCLA Molecular Diagnostics Laboratories via a clinically validated DNA Identity Test.

Retrospective exome data from 13 probands of the previously described UT adRP cohort of 265 families with a high likelihood of autosomal inheritance were analyzed for mutations in *ARL3*[28, 29]. An additional 47 probands from the UT adRP cohort and 34 adRP probands sent to the Laboratory for the Molecular Diagnosis of Inherited Eye diseases were also tested for mutations using fluorescent di-dexoy sequencing [30–32]. All probands had previously tested negative for mutations in known adRP genes. All six coding exons and a minimum of 20bp of flanking intronic sequence were tested for mutations (S3 Table).

## Results

A total of 1,787 rare coding variants were observed in at least one individual (annotated variants in S2 Table). Of these, 96 variants in 88 different genes passed all filtering criteria: observed as heterozygous or “no call” in all three individuals; MAF <0.1%, high sequencing quality; not in a gene known to cause an unrelated condition; not in a likely pseudogene (summary in Table 1; annotated variants list in S2 Table).

**Table 1:** Variants meeting various filter criteria. Maximum of 1000 Genomes and Exome Sequencing Project (ESP5400) used as minor allele frequency (MAF). “Problematic gene” is a gene which is known to be poorly assessed by exome sequencing, such as genes in the mucin family and HLA genes.

Filter	# of variants
Coding and MAF <1%	1,787
Het (or “no call”) in all 3	347
High quality (no flags, Q>500) autosomal	187
Very rare (MAF <0.1%)	135
Not in unrelated OMIM disease gene or “problematic gene”	96

Two variants were identified within genes known to be implicated in retinal degeneration, one each in *RP1L1* and *MFRP*. The c.3978_3979insGAA (p.Lys1326_Thr1327insGlu) variant in *RP1L1* (NM_178857.5) identified as heterozygous in all three individuals has not been previously observed in the general population or in individuals with retinal dystrophy. It is an in-frame insertion of a single amino acid. This variant was considered unlikely to be pathogenic for several reasons. As *RP1L1* has reduced penetrance [10] and a very high non-synonymous rare variant rate in the general population [33], the prior probability of identifying such a variant is quite high regardless of retinal phenotype. Additionally, the age of onset and retinal phenotype are not consistent with occult macular dystrophy, the clinical condition associated with *RP1L1*. At this time, this variant must be considered a variant of uncertain clinical significance. The c.1374G>T (p.Leu458Phe) variant in *MFRP* gene (NM_031433.2) is observed with a minor allele frequency of nearly 1% in Europeans [33], suggesting this variant is unlikely to cause highly penetrant disease in the heterozygous state.

**Fig 2:**
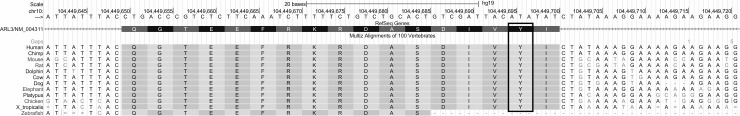
Evolutionary conservation of exon 5 of ARL3. Includes the tyrosine (Y) amino acid at position 90 (boxed), across vertebrate species. Representative set of 9 species shown using the UCSC Genome Browser.

Ranking of the 96 variants passing all filters was performed using clinical keywords. Manual interpretation of variants identified one strong candidate: a c.269A>G (p.Tyr90Cys) variant in the *ARL3* (ADP-ribosylation factor-like 3) gene (NM_004311.3, chr10:g.104449696T>C). It's gene product, the Arl3 protein, contains only 182 amino acids, making it a very small target for genetic variation. Likely due to the size of the gene, only 12 out of approximately 6,500 individuals (0.18%) in the Exome Sequencing Project dataset carry a rare heterozygous missense variant in *ARL3* [33]. None of these variants are within 10 amino acids of the p.Tyr90Cys variant identified in this study, suggesting that this interval may be relatively depleted of protein variation due to natural selection. All *in silico* prediction methods used supported the hypothesis that the p.Tyr90Cys variant is likely deleterious to the function of *ARL3* (Table 2). Of the vertebrate species with available data, all organisms (95 species spanning fish to primates) have a tyrosine at this aligned position in *ARL3*, indicating extremely strong evolutionary conservation of this amino acid [as per the Vertebrate Multiz Alignment & Conservation (100 Species) track of the UCSC genome browser accessed November 2015] (Fig 2). *In silico* analysis of protein-protein interactions between Arl3 and two of its major binding partners—RP2 and UNC119—predicts that the p.Tyr90Cys variant has a destabilizing effect on heterodimerization (Table 3).

**Table 2:** *In silico* prediction tests. Results for the p.Tyr90Cys variant in *ARL3*.

Test	Result
LRT Score (prediction)	0 (deleterious)
Polyphen2 HumDiv Score (prediction)	1 (probably damaging)
SIFT Score (prediction)	0 (probably damaging)
MutationTaster Score (Prediction)	0.99 (disease-causing)
PhyloP	2.1 (supports conservation)
PhastCons	15.5 (supports conservation)
GERP	5.3 (supports conservation)

**Table 3:** Protein-protein interaction predictions. Results for the p.Tyr90Cys variant in *ARL3*. Calculated using the web tool mCSM [27].

Arl3 Interaction Partner	PDB entry	ARL3 chain	mCSM ddG (Kcal/mol)	mCSM description
RP2/GDP-AlF4	3BH7	A	-0.719	Destabilizing
RP2/GppNHp	3BH6	A	-605	Destabilizing
UNC119A	4GOJ	A; B	-0.663; -0.741	Destabilizing; Destabilizing

The c.269A>G variant was confirmed as heterozygous in the three affected individuals and homozygous reference (“wild type”) in the unaffected grandparents by Sanger sequencing (S1 Fig). No rare coding variants in *ARL3* were found in an additional set of 94 families with RP.

## Discussion

*ARL3* encodes Arl3, a small GTPase which is a known binding partner of RP2 [34–36] and UNC119 [37]. Variants in *RP2* (retinitis pigmentosa 2) which encodes RP2 are associated with X-linked retinitis pigmentosa type 2 [MIM: 312600][38, 39]. Model organism work suggests that UNC119 function is critical to the visual system in mice [40]. Other known interaction partners include PDE6D, a protein whose gene is associated with primary cilia function [37] and disruption of which may cause Joubert Syndrome 22 [MIM: 615665][41]. Publicly available microarray data shows that *ARL3* is most highly expressed in the retina and pineal gland (GeneAtlas U133A, gcrma)[42]. These facts are consistent with functional studies demonstrating that the Arl3 protein is involved in protein trafficking of proteins to the outer segment of photoreceptor cells [34, 35, 43, 44]. A biochemical study of the RP2-null mouse model for RP suggests that the disease mechanism includes hyperactivity of Arl3 leading to mistrafficking of prenylated cargo in photoreceptor cells [45]. Given its expression pattern, binding partners, and biochemical, *ARL3* is a strong *a priori* candidate gene for retinitis pigmentosa in humans.

This candidacy is strengthened by the fact that the p.Tyr90Cys variant identified in the three affected individuals occurred *de novo* and transmitted with disease to a subsequent generation in a pedigree consistent with dominant RP. The failure to identify additional *ARL3* variants in our follow-up cohort suggests that even if the *ARL3* variant identified in our original family is truly causal for RP, such variants are responsible for only a small fraction of RP cases. Functional studies are merited to assess the biochemical impact of the p.Tyr90Cys variant on the Arl3 protein, and future genome-wide variant identification efforts of RP patients should consider *ARL3* as a gene of interest. Until functional studies are performed or additional families with *ARL3* variants are identified, this gene will remain a candidate for autosomal dominant retinitis pigmentosa.

## Supporting Information

S1 Fig Representative electropherogram results. Positive (top, proband) and negative (bottom, proband's mother) Sanger sequencing traces for the c.269C>A variant in *ARL3*. Arrows point to nucleotide of interest. Full electropherograms for all individuals in this family are available upon request.
(PDF)

S1 Table Description of data columns found in S3 Table. (XLS)

S2 Table All potentially causative rare coding variants identified in proband. For a description of column headers, please refer to S1 Table.
(XLS)

S3 Table Primer sequences. Used for *ARL3* coding sequence resequencing
(XLSX)
