# Supplementary material for: De Novo Occurrence of a Variant in ARL3 and Apparent Autosomal Dominant Transmission of Retinitis Pigmentosa
Source: PLoS One. 2016 Mar 10;11(3):e0150944. doi: 10.1371/journal.pone.0150944 (PMC4786330; doi:10.1371/journal.pone.0150944)
Supplement: S1 Fig — Positive (top, proband) and negative (bottom, proband's mother) Sanger sequencing traces for the c.269C>A variant in ARL3. Arrows point to nucleotide of interest. Full electropherograms for all individuals in this family are available upon request. (PDF) [file pone.0150944.s001.pdf]

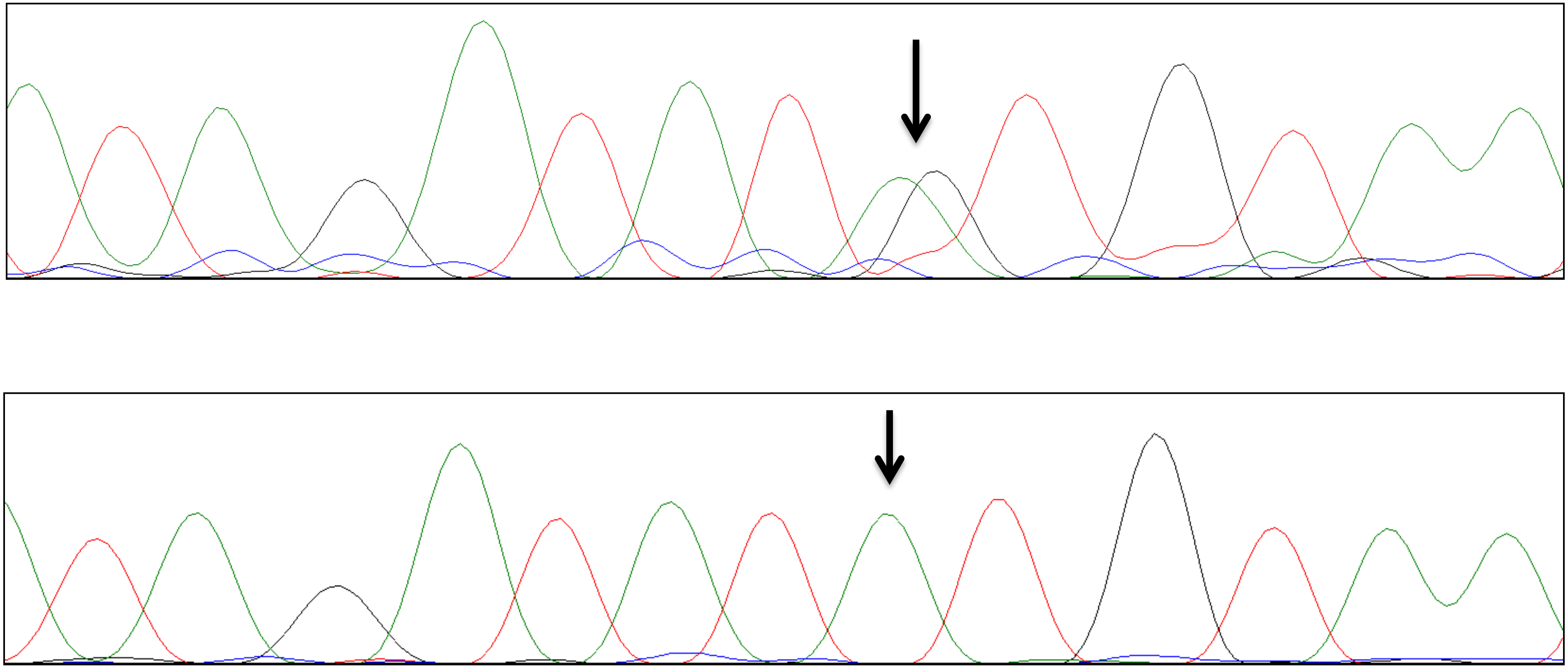

S1 Fig. Positive (top) and negative (bottom) Sanger sequencing traces for the c.269C>A (p.Tyr90Cys) variant in *ARL3.* Arrows point to nucleotide of interest.
